# Supplementary material for: Can High-Fidelity Patient Simulation Be Used for Skill Development in Junior Undergraduate Students: A Quasi-Experimental Study
Source: Healthcare (Basel). 2023 Aug 7;11(15):2221. doi: 10.3390/healthcare11152221 (PMC10418873; doi:10.3390/healthcare11152221)
Supplement: Supplementary file 1 [file healthcare-11-02221-s001.zip › healthcare-2506011-supplementary.pdf]

Table S1. Differences between standard HFPS and HFPS with structured guideline

Stages	Characteristics	Standard HFPS (An educator was responsible for this group)	HFPS with structured guideline (Another educator was responsible for this group)
Pre-simulation	Pre-simulation study	• No consensus.<br>• Students may receive preparatory materials for simulation provided by the educator.	Pre-simulation reading materials will be sent to students. Students are required to read the materials before simulation lab.
	Simulated case scenario	The course coordinator will only upload the case scenario with brief information to students	The case scenario information and condition in 3 stages (for 3 small groups individually) will be sent to the students.
	Pre-briefing of the simulation lab and the instruction of the simulation including roles and responsibilities	No consensus and depending on the responsible lab teachers.	The educator will introduce the simulation lab environment as well as students' and educator's roles and responsibilities.
During simulation	Performing in HFPS	• Students are divided into 3 small groups and expected to perform nursing care and skill practice to the simulated case.<br>• Students are expected to apply their learned knowledge and skills	• Students are divided into 3 small groups and expected to perform nursing care and skill practice to the simulated case.<br>• Students are expected to apply their learned knowledge and skills.
	Time period of simulation performance	15 to 20 minutes	20 minutes
	Behavior/ tasks of students who are observers.	• No consensus<br>• Self-manage or some tasks will be given by the educator	• Students are required to record the areas that have been well performed and that must be improved regarding their observation of the students in HFS.<br>• Students will have the guiding questions to let them understand more about the roles of players.
Post-simulation	Debriefing	Mainly students will listen to the feedback from the educators.	• All students will be required to give comments on the performance of the group in HFS.<br>• Individual students will be requested to reflect their performance.<br>• The educator will give comments on overall student performance for improvement.
